# A tree peony RING-H2 finger protein, PsATL33, plays an essential role in cold-induced bud dormancy release by regulating gibberellin content

**DOI:** 10.3389/fpls.2024.1395530

**Published:** 2024-06-03

**Authors:** Yanxiang Mao, Yanping Yuan, Yeshen Gao, Lingling Zeng, Siyu Fan, Jianrang Luo, Daoyang Sun

**Affiliations:** ^1^ College of Landscape Architecture and Arts, Northwest A&F University, Yangling, Shaanxi, China; ^2^ National Engineering Technology Research Center for Oil Peony, Northwest A&F University, Yangling, Shaanxi, China

**Keywords:** tree peony, bud dormancy, PsATL33, abscisic acid, gibberellins

## Abstract

Bud dormancy is crucial for woody perennial plants to resist low-temperature stress in winter. However, the molecular regulatory mechanisms underlying bud dormancy release are largely unclear. Here, a tree peony (*Paeonia suffruticosa*) transcript *ARABIDOPSIS TOXICOS EN LEVADURA 33* (*PsATL33*), encoding a RING-H2 finger protein, was selected from previously generated RNA sequencing data of chilling-treated buds. The objective of this study is to investigate the role of PsATL33 in the regulation of cold-induced bud dormancy release. Subcellular localization assay revealed that PsATL33 was localized to the nucleus and plasma membrane. Reverse transcription–quantitative PCR analysis showed that *PsATL33* was dramatically upregulated during cold-triggered bud dormancy release. Exogenous treatments with gibberellin (GA_3_) increased, but abscisic acid (ABA) inhibited the transcription of *PsATL33*. Ectopic transformation assay indicated that overexpression of *PsATL33* in petunia promoted seed germination, plant growth, and axillary bud break. Silencing of *PsATL33* in tree peony through virus-induced gene silencing assay delayed bud dormancy release. tobacco rattle virus (TRV)-*PsATL33*-infected buds exhibited reduced expression levels of dormancy break-related genes *EARLY BUD-BREAK 1* (*PsEBB1*) and *CARBOXYLESTERASE 15* (*PsCXE15*). Silencing of *PsATL33* decreased the accumulation of bioactive GAs, GA_1_ and GA_3_, rather than ABA. Transcript levels of several genes involved in GA biosynthesis and signaling, including *GA20-OXIDASE 1* (*PsGA20ox1*), *GA3-OXIDASE 1* (*PsGA3ox1*), *PsGA3ox3*, *GA2-OXIDASE 1* (*PsGA2ox1*), and *GA-INSENSITIVE 1A* (*PsGAI1A*), were changed by *PsATL33* silencing. Taken together, our data suggest that PsATL33 functions as a positive regulator of cold-induced bud dormancy release by modulating GA production.

## Introduction

The dormancy represents one of the most adaptive responses of plants to cope with cold stress in winter (Cooke et al., 2012). The stages of bud dormancy can be classified into paradormancy, endodormancy, and ecodormancy (Gillespie and Volaire, 2017). Of them, the state of shoot growth cessation and bud set is referred to as endodormancy (Sasaki et al., 2011). Low temperature and short-day photoperiod can induce plant growth arrest and bud endodormancy (Yang et al., 2021). For temperature-sensitive perennials, the buds must achieve adequate exposure to low temperatures to fulfill their cold requirements (Anderson et al., 2010), which initiates bud dormancy release and break in spring (Fadón et al., 2020). It is also a crucial developmental step that affects plant growth and flowering (Yamane et al., 2023).

Endogenous hormones are critical regulators of the bud endodormancy process, especially gibberellins (GAs) and abscisic acid (ABA) (Yang et al., 2019). It is well known that GAs play an important role in chilling-induced bud dormancy release (Schrader et al., 2004). GA content has been shown to increase after chilling treatment. Bud burst only occurred when sufficient levels of GA_4_ were present in poplar buds (Rinne et al., 2011). GA_4_ treatment accelerated the rate of bud burst in Japanese apricot (Zhuang et al., 2013). ABA content varies during bud dormancy establishment, maintenance, and release (Liu and Sherif, 2019). ABA functions as a crucial signal in response to short-day photoperiod during the harshest seasons (Zheng et al., 2015). Application of ABA led to plant growth cessation and bud dormancy development in various birch ecotypes (Li et al., 2003a). ABA production reached the maximum levels in potato tubers that were completely dormant (Destefano-Beltrán et al., 2006). Increased ABA levels were found at the onset of bud dormancy in sweet cherry, followed by a decrease during the transition from endodormancy to ecodormancy (Vimont et al., 2021). In flower buds of sweet cherry, the ratio of ABA/GA_3_ increased during dormancy induction and decreased during dormancy release (Wang et al., 2017b).

In recent years, significant progress has been made to reveal the regulatory mechanisms underlying cold-induced bud dormancy release (Yang et al., 2021). DORMANCY-ASSOCIATED MADS-BOX (DAM) and SHORT VEGETATIVE PHASE-LIKE (SVL) proteins, belonging to the MADS-box transcription factor family, are considered critical regulators of the bud dormancy process (Falavigna et al., 2019). In hybrid aspen, an ortholog of SVL negatively regulated the GA pathway to promote bud dormancy (Singh et al., 2019). In kiwifruit, SVP2 played a pivotal role in preventing premature bud break during dormancy (Wu et al., 2017c). Ectopic overexpression of *MdDAMb* and *MdSVPa* in apple also resulted in delayed bud break (Wu et al., 2017b). In Japanese apricot, PmRGL2 functioned as a negative regulator of bud dormancy by affecting the transcription of several GA biosynthetic and signaling genes (Lv et al., 2018). A recent report showed that PpMAPK6 accelerated the degradation of PpDAM6 through phosphorylation and promoted the dormancy release of peach flower buds (Zhang et al., 2023). However, few studies have focused on the regulation of bud dormancy in woody ornamental plants.

Tree peony (*Paeonia* section *Moutan* DC.), belonging to the family Paeoniaceae, is a woody perennial shrub with great ornamental value (Guo et al., 2020). Tree peony bud is a typical compound bud whose dormancy is known as endodormancy (Xin et al., 2019). Bud dormancy is a constraining factor for a successful forcing culture in the tree peony industry. Therefore, the elucidation of molecular mechanisms of tree peony bud dormancy release is required. It has been revealed that a sufficient chilling duration is required to promote bud dormancy release in tree peony. The temperature ranging from 0°C to 4°C is the most frequently used to break bud dormancy (Huang et al., 2008). GAs have been suggested as the primary signals in the chilling-induced bud dormancy release of tree peony (Gai et al., 2013). In particular, exogenous GA_3_ application resulted in faster bud burst, shoot growth, and flowering (Zhang et al., 2021). It was found that PsBG6 responded to low temperatures and regulated GA-induced bud dormancy release in tree peony (Gao et al., 2021). *PsRGL1*, encoding a DELLA protein, played an important role in the regulation of bud dormancy by suppressing GA signaling (Gao et al., 2023). Moreover, artificial chilling and exogenous GA treatments are two common methods to break tree peony bud dormancy. Accordingly, bud break is an economically and environmentally important process in tree peony, but its molecular regulatory mechanisms are not fully understood.

In previous studies, we performed RNA sequencing (RNA-Seq) analysis of tree peony buds during chilling-induced dormancy release (Yuan et al., 2024). A large number of differentially expressed genes were identified from RNA-Seq data. Given the importance of ARABIDOPSIS TOXICOS EN LEVADURAs (ATLs) in plant response to environmental stresses (Wu et al., 2023), one upregulated transcript, *PsATL33*, was selected for functional characterization. We hypothesized that this upregulation may indicate a crucial role of PsATL33 in the regulation of cold-induced tree peony bud dormancy release. The experiments presented here were conducted to test this hypothesis.

## Materials and methods

### Plant materials and growth conditions

One-year-old grafted tree peony plants (*Paeonia suffruticosa* ‘Yulouchun’), obtained from Luhe Tree Peony Planting Professional Cooperative (Heze, China), were used as the main experimental materials in this study. The plants were planted in plastic pots (16-cm height, 14-cm top diameter, and 11-cm bottom diameter) filled with a soil mixture containing peat moss and perlite (2:1, by vol.) in late September. Each pot was irrigated with 300 mL of tap water containing an appropriate concentration of complex fertilizer (N:P:K = 2:1:1, by wt.) once per week. They were kept in an outdoor environment (a temperature range from 12°C to 21°C, relative humidity of 52%–80%, and natural photoperiod) until early November when the bud dormancy was completely established. The plants were transferred to a cold chamber at 2°C for chilling treatment. The apical buds treated with low temperature at intervals (0 days, 5 days, 10 days, 15 days, 20 days, 25 days, and 30 days) were used for gene expression analysis. The buds at 25 days of chilling treatment, near the time of a complete endodormancy release, were used for a tobacco rattle virus (TRV)-based virus-induced gene silencing (VIGS) experiment. Petunia seeds (*Petunia hybrida* ‘Mitchell Diploid’), obtained from Goldsmith Seeds (Gilroy, CA, USA), were sown in a tray containing the same soil mixture. The leaflets were harvested as the explants for *Agrobacterium*-mediated stable transformation (Ji et al., 2023). Wild-type (WT) and transgenic petunia plants were grown in the plastic pots (11-cm height, 11-cm top diameter, and 9-cm bottom diameter) and irrigated as mentioned above. Tree peony plants upon VIGS assay and petunia plants were maintained in a growth chamber at 22°C day/20°C night with a 16-h light/8-h dark photoperiod.

### Isolation and identification of *PsATL33*

Based on the RNA-Seq data of tree peony buds, the cDNA sequence of *PsATL33* containing a 483-bp coding region was isolated. The translation of nucleotides into amino acids was conducted using the ExPASy tool (http://web.expasy.org/translate/). Phylogenetic tree analysis was carried out using MEGA4 software (version 4.0.2). The RING-H2 conserved domain was identified according to a previous report (Serrano et al., 2006). The modeling process of the RING-H2 domain was performed using a Modeling server (version 9.20) based on the sequence alignment. A similar protein structure (Protein Data Bank: 1X4J) of PsATL33 was used as the template. The model was evaluated through Discrete Optimized Protein Energy (DOPE) values and GA 341 scores and finally visualized using the PyMOL tool (version 2.5.4).

### Subcellular localization assay

The coding region of *PsATL33* was amplified with the stop codon removed to construct the pCAMBIA2300-*PsATL33*–green fluorescent protein (GFP) fusion expression vector. The expression vector containing *PIP2A*-mCherry was used as the control to mark the plasma membrane. The fusion expression vectors were transformed into *Agrobacterium tumefaciens* GV3101 through the freeze–thaw method. The *Agrobacteria* transformed with *PsATL33*-GFP and *PIP2A*-mCherry were mixed in equal proportion and injected into 4-week-old *Nicotiana benthamiana* leaves. The reagent 4′,6-diamidino-2-phenylindole (DAPI) was used to mark the nucleus. The fluorescence was observed under a laser scanning confocal microscope (TCS SP8 SR; Leica, Wetzlar, Germany). GFP, mCherry, and DAPI were excited using 488-, 561-, and 405-nm lasers and detected after passing through 500–560-nm, 590–620-nm, and 410–492-nm band-pass filters, respectively.

### Exogenous hormone and abiotic stress treatments

To examine the effects of exogenous hormones and abiotic stresses on expression profiles of *PsATL33*, tree peony buds before artificial chilling treatment were used. For exogenous hormone treatments, the buds were sprayed with solutions containing 100 μM GA_3_, 100 μM ABA, 100 μM salicylic acid (SA), 50 μM ethephon (ETH), and 100 μM methyl jasmonate (MeJA). The control buds were treated with deionized water. For abiotic stress treatments, the plants were irrigated with 20% PEG6000 and 100 mM NaCl or placed in a warm room (37°C) and a cold room (−4°C). For each case, the buds with three biological replicates were collected at 0 h, 6 h, 12 h, 24 h, 36 h, and 48 h after treatment. They were frozen in liquid nitrogen and stored at −80°C.

### Reverse transcription–quantitative PCR assay

Total RNAs of tree peony buds and petunia leaves were extracted using RNAprep Pure Plant Kit (Tiangen, Beijing, China). To remove DNA contamination, RNA samples were treated with RNase-free DNase I (Promega, Madison, WI, USA) at 37°C for 30 min. RNA concentration and quality were evaluated via 1.2% agarose gel electrophoresis and a spectrophotometer (NanoDrop, Wilmington, DE, USA). First-strand cDNA was synthesized from 2–5 µg of RNA samples using PrimeScript RT reagent (Takara, Otsu, Shiga, Japan). Reverse transcription–quantitative PCR (RT-qPCR) analysis was performed using the synergy brands (SYBR) Green Reagent in a LightCycler480 Real-Time PCR System (Roche Diagnostic, Basel, Switzerland). *PsActin* and *PhEF1α* were used as the reference genes in tree peony and petunia, respectively. The primer pairs were designed using Primer3 Input (version 0.4.0) and shown in **Supplementary Table S1**. Relative expression levels of the genes were calculated as previously described (Livak and Schmittgen, 2001). Three biological replicates were used in this experiment.

### Detection of gibberellin and abscisic acid contents

The samples were ground into powder in liquid nitrogen and extracted with 80% (v/v) methanol and 1 mM butyl hydroxytoluene. The extract was transferred into a tube with 20 mg of polyvinylpolypyrrolidone and fully mixed. The mixture was centrifuged at a relatively low temperature at 5,000 rpm for 20 min. Under the condition of 40°C, the extract was almost concentrated into a water phase containing ammonia. After filtration with a 0.45-µm filter, the samples were dried by vacuum freezing. The particles were then dissolved in 50% (v/v) methanol and analyzed through high-performance liquid chromatography using an Agilent chromatograph (Model 1100, Agilent Technologies, Santa Clara, CA, USA). The detection wavelength used in this assay was 210 to 280 nm, and the flow rate was 1 mL/min. The standard hormones were purchased from Sigma-Aldrich (St Louis, MO, USA). The peak areas were analyzed to quantify the levels of bioactive GAs and ABA. Three biological replicates were used for each hormone measurement.

### Generation of transgenic petunia plants

The coding region of *PsATL33* was PCR-amplified and cloned into a modified pCAMBIA1300 vector to generate the overexpression construct (Sun et al., 2020). The recombinant plasmid was transformed into *A. tumefaciens* GV3101. The leaf disc method was used for stable genetic transformation of petunia plants (Yuan et al., 2024). Briefly, young leaves from petunia ‘Mitchell Diploid’ plants were collected and cut into 1 cm × 1 cm leaf discs, which were infected with *Agrobacteria* harboring the recombinant plasmids. The inoculated leaves were placed on a co-cultivation medium at room temperature for 2 days and then transferred to a fresh regeneration medium for the selection of positive transformants. Regular PCR amplification was conducted when the plants reached the four-leaf stage to confirm the integration of *PsATL33*. Transcript abundances of *PsATL33* in leaves from transgenic petunia lines were examined by RT-qPCR. Three lines with higher transcription of *PsATL33* were selected for functional investigation.

### Virus-induced gene silencing assay

To generate the TRV-*PsATL33* construct, a 347-bp fragment of *PsATL33* was PCR-amplified and introduced into the TRV2 vector between *Kpn*I and *Xho*I sites. TRV2 empty vector and TRV-*PsATL33* plasmids were transformed into *A. tumefaciens* GV3101. The inoculum was prepared according to a previous description (Mao et al., 2022). The transformed *Agrobacteria* were cultured in Luria-Bertani (LB) media (40 mg/L kanamycin, 20 mg/L gentamicin, 10 mM MES, and 20 μM acetosyringone) at 28°C for 48 h. When the OD600 reached 2.0, the cells were harvested and resuspended in the infiltration buffer (10 mM MgCl_2_, 10 mM MES, and 200 μM acetosyringone). After a gentle shaking for 3 h, *Agrobacterium* cultures bearing TRV1 and TRV2 empty vectors or TRV-*PsATL33* were mixed together in equal volumes. The bacterial solution was used to inoculate tree peony buds under a vacuum pressure at 0.7 MPa for 20 min. The inoculated plants were transplanted into the soil mixture and maintained in a cold room at 10°C for 2 days, which would help increase viral accumulation. Next, the plants were transferred to a growth chamber for phenotype observation. Three tree peony plants were used for each inoculation.

### Freezing tolerance assay

To investigate the function of PsATL33 in response to freezing stress, tree peony plants inoculated with TRV empty vector and TRV-*PsATL33* were placed in a cold room at −4°C with light illumination. A normal condition at 22°C was used as the control. After cold treatment, the survival rate of tree peony buds was recorded at 0 and 6 h post-freezing. The buds were collected at the same time points for measurement of malondialdehyde (MDA) content and ion leakage rate. Three biological replicates were used with a pool of three seedlings per replicate. The detection of MDA content was performed as previously described (Shin et al., 2012). In brief, the bud tissues were homogenized with 0.1% (w/v) trichloroacetic acid on ice. The mixture was centrifuged at 12,000 rpm for 10 min at 4°C. The resulting supernatant was evenly blended with 0.25% (w/v) thiobarbituric acid. The mixture was heated in a water bath at 95°C for 10 min and cooled rapidly to 4°C for further centrifugation at 4,500 rpm for 10 min. The supernatant was detected by measuring A_450_, A_532_, and A_600_ values, with deionized water being the blank control. MDA content was analyzed based on the following formula: 6.45 (A_532_ − A_600_) − 0.56 A_450_. The ion leakage rate was measured following a previously described method (Wu et al., 2017a). First, the buds were soaked in 0.4 M mannitol at 20°C for 3 min, and the initial conductivity was measured using a meter (Leici, Shanghai, China). The samples were heated in a water bath at 85°C for 20 min, and the total conductivity was measured thereafter. The ion leakage rate was determined as the percentage of initial conductivity to total conductivity. Three biological replicates were used for each measurement.

### Statistical analysis

All experiments reported here were performed using three biological replicates. Statistical significance was evaluated through Student’s *t*-test at *p*-values <0.05 and <0.01 using JMP software (version 11.0; SAS Institute Inc., Cary, NC, USA).

## Results

### PsATL33 contains a RING-H2 domain and is localized into nucleus and plasma membrane

**Figure 1:**
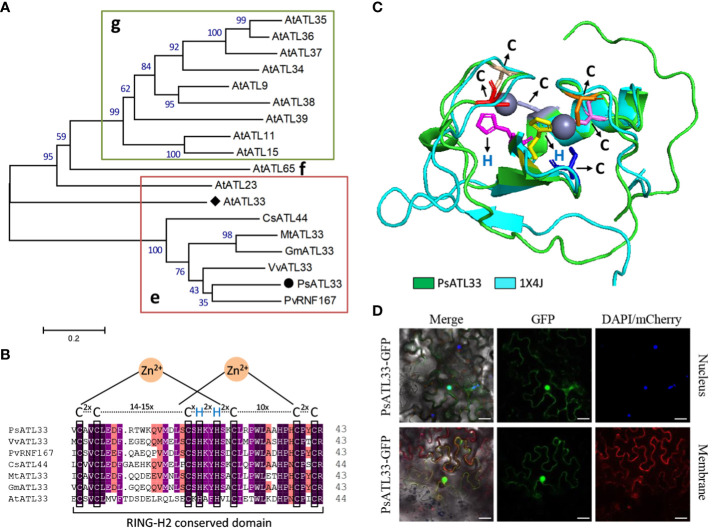
Amino acid sequence and subcellular localization analysis of PsATL33. **(A)** Phylogenetic tree of PsATL33 with its similar proteins from *Vitis vinifera* (VvATL33), *Pistacia vera* (PvRNF167), *Glycine max* (GmATL33), *Medicago truncatula* (MtATL33), *Camellia sinensis* (CsATL44), and *Arabidopsis thaliana* (AtATL33 and other ATLs) belonging to groups e, f, and g of ATL family. Bootstrap values are expressed as a percentage of 1,000 replicates and shown at branch nodes. PsATL33 is marked by a solid circle. AtATL33 is denoted by a solid diamond. **(B)** Alignment of RING-H2 conserved domains within PsATL33 and its homologous proteins. The key cysteine **(C)** and histidine (H) residues are boxed. **(C)** Protein modeling of PsATL33 in superimposition with its similar protein structure 1X4J. The C and H residues are shown as sticks with different colors. **(D)** Subcellular localization of PsATL33 in *Nicotiana benthamiana* leaves using PsATL33-GFP fusion. DAPI and PIP2A-mCherry were used to mark the nucleus and plasma membrane, respectively. Scale bars = 20 μm.

To investigate the molecular mechanisms of bud dormancy break in tree peony, a 783-bp transcript encoding a putative RING-H2 finger protein, designated *PsATL33*, was identified from transcriptome data of chilling-treated tree peony buds (Yuan et al., 2024). Sequence analysis revealed that its cDNA contains a complete coding region of 483 bp, encoding a polypeptide of 161 amino acids (**Supplementary Figure S1**). PsATL33 was phylogenetically close to AtATL33 from *Arabidopsis thaliana*, PvRNF167 from *Pistacia vera*, and other ATL33s from *Vitis vinifera*, *Glycine max*, *Medicago truncatula*, and *Camellia sinensis* (**Figure 1A**). The C-terminuses of these amino acid sequences shared a conserved RING-H2 domain. The characteristic sequence of this domain is Cys-2X-Cys-14–15X-Cys-1X-His-2X-His-2X-Cys-10X-Cys-2X-Cys (X represents any amino acids except Cys and His), which belongs to the C3H2C3-type RING-H2 domain (**Figures 1B, C**). To study the subcellular localization of PsATL33, the fusion protein PsATL33-GFP was transiently expressed in tobacco leaves. DAPI staining and co-expression with PIP2A-mCherry were used as nucleus and plasma membrane markers, respectively. The results indicate that PsATL33 is localized into the nucleus and plasma membrane (**Figure 1D**).

### *PsATL33* is upregulated during chilling-induced bud dormancy release

**Figure 2:**
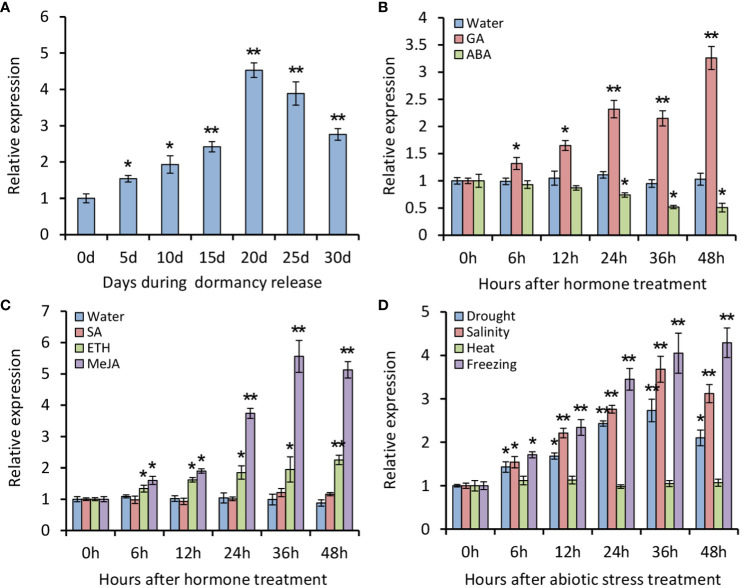
Expression analysis of *PsATL33* during bud dormancy release and in response to exogenous hormones and abiotic stresses. **(A)** Reverse transcription–quantitative PCR (RT-qPCR) analysis of expression levels of *PsATL33* in tree peony buds during chilling-induced dormancy release. The samples were collected on different days (d) after chilling treatment. RT-qPCR analysis of expression levels of *PsATL33* in the buds at different hours (h) after treatments with 100 μM gibberellin (GA_3_), 100 μM abscisic acid (ABA) **(B)**, 100 μM salicylic acid (SA), 50 μM ethephon (ETH), and 100 μM methyl jasmonate (MeJA) **(C)**. Treatment with distilled water was used as the control. **(D)** RT-qPCR analysis of expression levels of *PsATL33* in the buds upon exposure to drought, salinity (NaCl), heat (37°C), and freezing (−4°C) at intervals. *PsActin* was used as an internal control. Error bars represent standard error of the mean from three biological replicates. Asterisks indicate statistical significance as calculated by Student’s *t*-test (^∗^ *p* < 0.05, ^∗∗^ *p* < 0.01).

To understand expression patterns of *PsATL33*, RT-qPCR analysis was performed using tree peony buds. It was found that expression levels of *PsATL33* initially increased during chilling-induced bud dormancy release, followed by a slight decrease in the last 10 days (**Figure 2A**). Transcription of *PsATL33* increased significantly after GA_3_ treatment but decreased after ABA treatment (**Figure 2B**). The effects of several stress-associated hormones and abiotic stresses on *PsATL33*’s expression were also examined. ETH and MeJA treatments resulted in a significant increase in the expression of *PsATL33*, but there was no significant change after SA treatment (**Figure 2C**). In addition, transcript levels of *PsATL33* increased following treatments with drought, salinity, and freezing rather than heat (**Figure 2D**). These data indicate that *PsATL33* is responsive to dormancy- and stress-related hormonal or environmental signals.

### Overexpression of *PsATL33* promotes petunia seed germination, plant growth, and axillary bud break

**Figure 3:**
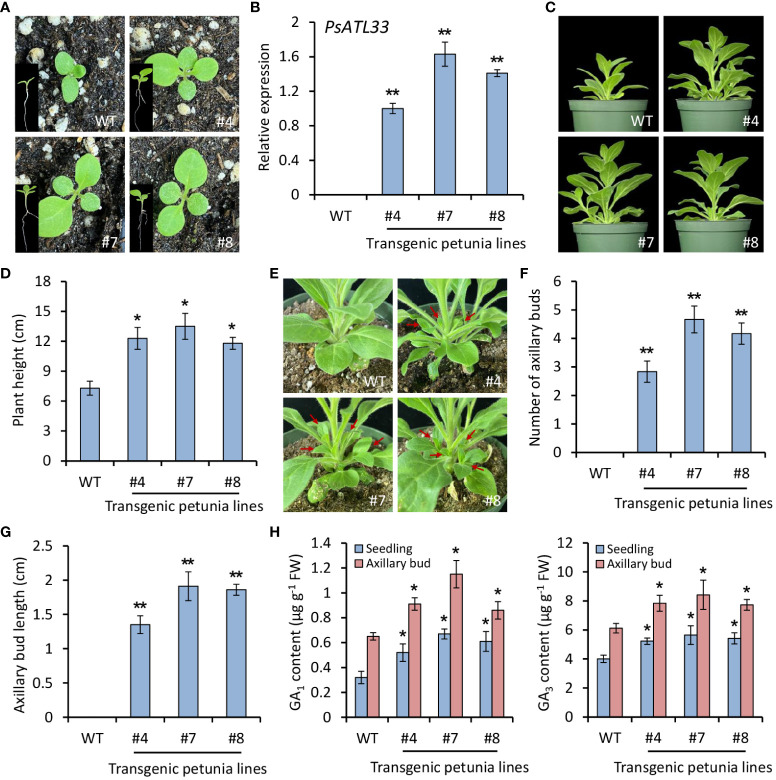
Overexpression of *PsATL33* promotes seed germination, plant growth, and axillary bud break in petunia. **(A)** Representative phenotypes of germinating seeds from wild-type (WT) and *PsATL33*-overexpressing transgenic petunia lines (#4, #7, and #8) at 12 days after sowing. The insets are the whole phenotypes of petunia seedlings. **(B)** Reverse transcription–quantitative PCR (RT-qPCR) analysis of expression levels of *PsATL33* in the seedlings from WT and transgenic petunia lines. *PsActin* was used as an internal control. **(C)** Representative phenotypes of WT and *PsATL33*-overexpressing transgenic petunia plants at 40 days after sowing. **(D)** Plant height of transgenic petunia lines in comparison to WT. **(E)** Representative phenotypes of axillary buds from WT and transgenic petunia lines at 40 days after sowing. Red arrows indicate the axillary bud outgrowth. Axillary bud number **(F)** and length **(G)** of transgenic petunia lines compared to WT. **(H)** Accumulation of bioactive gibberellins (GA_1_) and (GA_3_) in the seedlings and axillary buds of transgenic petunia lines. The seedlings at 12 days and axillary buds at 40 days after sowing were collected for measurement. Expression levels were standardized to *PhEF1α*. Error bars represent standard error of the mean from three biological replicates. Asterisks indicate statistical significance as determined by Student’s *t*-test (^∗^ *p* < 0.05, ^∗∗^ *p* < 0.01).

To study the function of PsATL33 in bud dormancy, we carried out a heterologous transformation experiment in petunia (*P. hybrida*). Compared with WT plants, *PsATL33*-overexpressing transgenic plants exhibited accelerated seed germination (**Figure 3A**). RT-qPCR results confirmed the constitutive expression of *PsATL33* in transgenic petunia lines (**Figure 3B**). A shorter seed germination time was found in transgenic lines overexpressing *PsATL33* than that in the WT line (**Supplementary Figure S2A**). Overexpression of *PsATL33* resulted in increased petunia plant height and internode length in the following growth periods (**Figures 3C, D**; **Supplementary Figure S2B**). Its overexpression also led to enlarged leaf size in petunia (**Supplementary Figure S3**). In addition, *PsATL33*-overexpressing transgenic plants displayed promoted growth of axillary buds (**Figure 3E**). The number and length of axillary buds in transgenic petunia lines were significantly higher than those in WT control (**Figures 3F, G**). Increased production of bioactive GAs, GA_1_ and GA_3_, was found in *PsATL33*-overexpressing transgenic lines (**Figure 3H**). These findings suggest that overexpression of *PsATL33* promotes the dormancy release of petunia seeds and axillary buds.

### Silencing of *PsATL33* inhibits tree peony bud dormancy release

**Figure 4:**
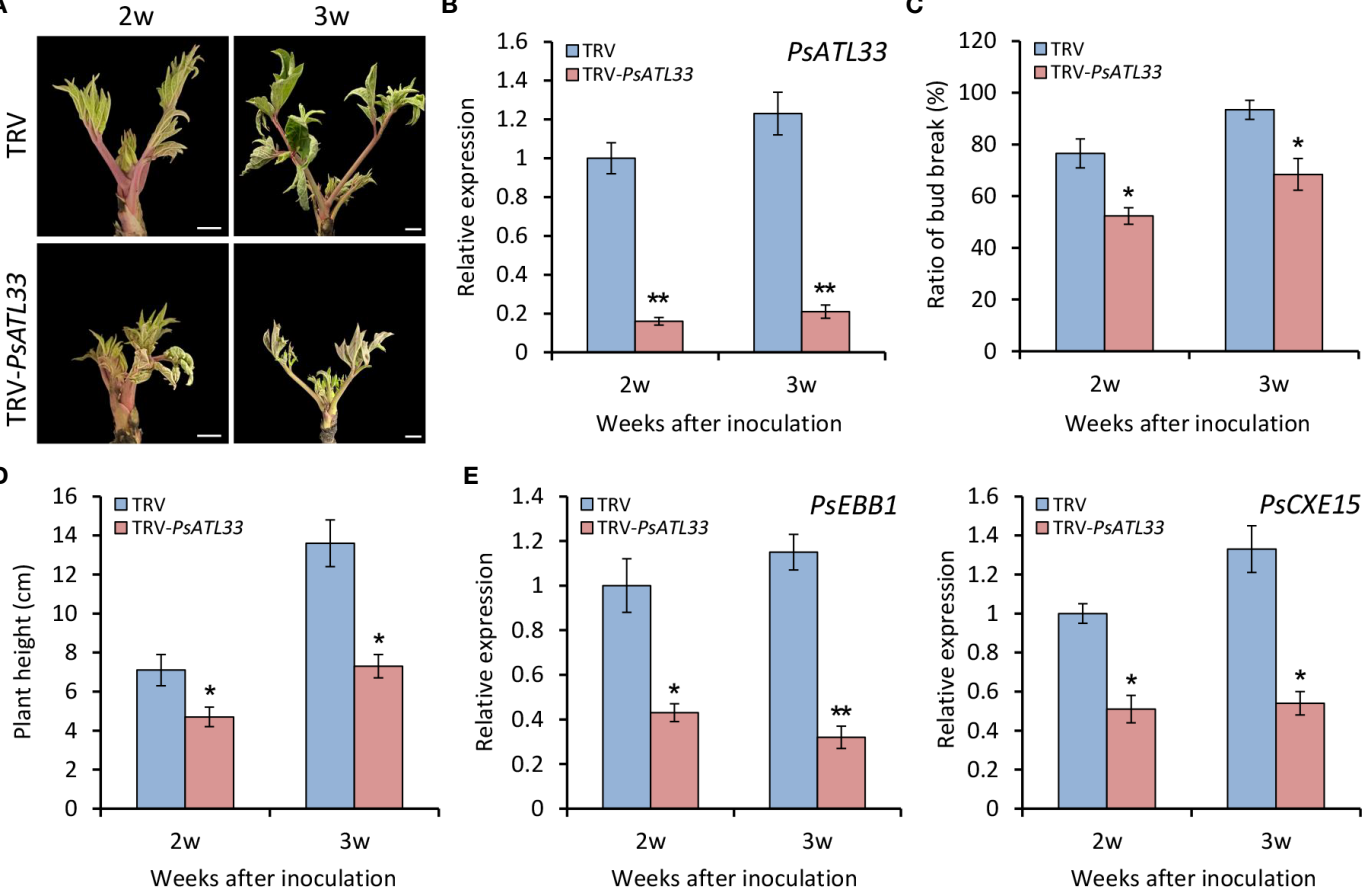
Silencing of *PsATL33* inhibits bud dormancy release in tree peony. **(A)** Representative phenotypes of sprouting buds from tree peony plants inoculated with tobacco rattle virus (TRV) empty vector and TRV-*PsATL33* at 2 and 3 weeks (w) after inoculation. The chilling-treated buds for 25 days were used in the virus-induced gene silencing (VIGS) assay. Scale bars = 0.8 cm. **(B)** Reverse transcription–quantitative PCR (RT-qPCR) analysis of expression levels of *PsATL33* in the buds inoculated with various TRV constructs at 2 and 3 w after inoculation. Bud break rate **(C)** and plant height **(D)** of TRV empty vector- and TRV-*PsATL33*-infected tree peony plants at different time points. **(E)** RT-qPCR analysis of expression levels of dormancy break-associated genes *PsEBB1* and *PsCXE15* in the buds inoculated with various TRV constructs at different time points. *PsActin* was used as an internal control. Error bars represent standard error of the mean from three biological replicates. Asterisks indicate statistical significance as evaluated by Student’s *t*-test (^∗^ *p* < 0.05, ^∗∗^ *p* < 0.01).

To further verify the function of PsATL33 in bud dormancy, we performed a TRV-based VIGS experiment. Compared with empty vector control, tree peony plants inoculated with TRV-*PsATL33* showed delayed bud break and growth at 2 and 3 weeks after inoculation (**Figure 4A**). RT-qPCR results showed that expression levels of *PsATL33* in the buds inoculated with TRV-*PsATL33* were significantly reduced in comparison to the control, suggesting a successful silencing of *PsATL33* in the buds (**Figure 4B**). Consistent with the phenotypes, the *PsATL33*-silenced buds showed significantly reduced bud break rate and plant height (**Figures 4C, D**). Transcript levels of two bud dormancy break-associated genes, *EARLY BUD-BREAK 1* (*PsEBB1*) (Zhang et al., 2021) and *CARBOXYLESTERASE 15* (*PsCXE15*) (Huang et al., 2008), were dramatically lower in *PsATL33*-silenced buds than that in empty vector control (**Figure 4E**), indicating that silencing of *PsATL33* inhibits tree peony bud dormancy release.

### Silencing of *PsATL33* affects the production of gibberellins rather than abscisic acid

**Figure 5:**
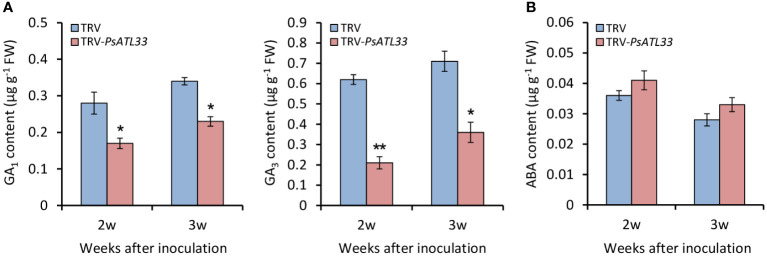
Silencing of *PsATL33* decreases gibberellin content in tree peony buds. Accumulation of bioactive gibberellins (GA_1_) and (GA_3_) **(A)** and abscisic acid (ABA) **(B)** in the buds infected with tobacco rattle virus (TRV) empty vector and TRV-*PsATL33* at 2 and 3 weeks (w) after inoculation. Error bars represent standard error of the mean from three biological replicates. Asterisks indicate statistical significance as calculated by Student’s *t*-test (^∗^ *p* < 0.05, ^∗∗^ *p* < 0.01).

Given the essential roles of GAs and ABA in the regulation of bud dormancy, we detected their contents in tree peony buds upon VIGS assay. In comparison to empty vector control, the accumulation of bioactive GAs, GA_1_ and GA_3_, in TRV-*PsATL33*-infected buds was significantly reduced at different weeks after infiltration, and notably, the decrease of GA_3_ accumulation was more significant than that of GA_1_ (**Figure 5A**). However, no significant alteration in ABA accumulation was found in the buds infiltrated with TRV-*PsATL33* (**Figure 5B**).

**Figure 6:**
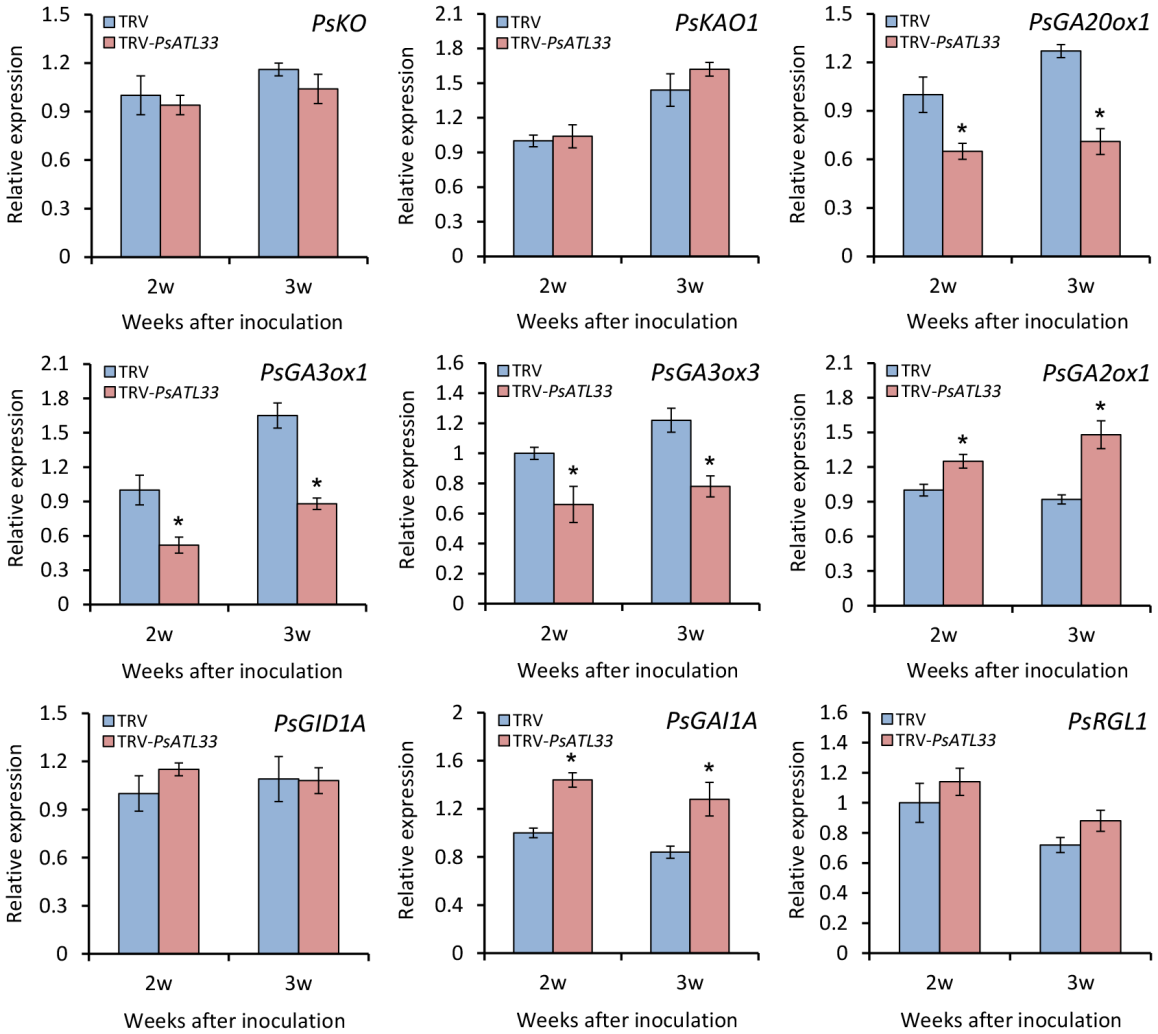
Silencing of *PsATL33* downregulates gibberellin biosynthesis and signaling-related genes in tree peony buds. Reverse transcription–quantitative PCR (RT-qPCR) analysis of expression levels of gibberellin biosynthesis- and signaling-related genes, including *PsKO*, *PsKAO1*, *PsGA20ox1*, *PsGA3ox1*, *PsGA3ox3*, *PsGA2ox1*, *PsGID1A*, *PsGAI1A*, and *PsRGL1*, in the buds inoculated with tobacco rattle virus (TRV) empty vector and TRV-*PsATL33* at 2 and 3 weeks (w) after inoculation. *PsActin* was used as an internal control. Error bars represent standard error of the mean from three biological replicates. Asterisks indicate statistical significance as determined by Student’s *t*-test (^∗^ *p* < 0.05).

To further study the role of PsATL33 in mediating the GA pathway, we analyzed the transcription of some genes related to GA biosynthesis and signal transduction in the buds. RT-qPCR analysis showed that expression levels of GA biosynthetic genes *GA20-OXIDASE 1* (*PsGA20ox1*), *GA3-OXIDASE 1* (*PsGA3ox1*), and *PsGA3ox3* decreased significantly in *PsATL33*-silenced buds compared to the control, whereas transcript abundances of GA catabolic gene *GA2-OXIDASE 1* (*PsGA2ox1*) and GA signal transduction repressor *GA-INSENSITIVE 1A* (*PsGAI1A*) increased (**Figure 6**). No significant difference in transcript levels of other GA pathway-related genes, *ENT-KAURENE OXIDASE* (*PsKO*), *ENT-KAURENOIC ACID OXIDASE 1* (*PsKAO1*), *GA-INSENSITIVE DWARF 1A* (*PsGID1A*), and *RGA-LIKE 1* (*PsRGL1*), was observed in the buds infected with empty vector and TRV-*PsATL33* (**Figure 6**). These results demonstrate that PsATL33 affects bud dormancy release by modulating GA production.

## Discussion

Bud dormancy is an effective strategy for woody perennial plants to resist cold weather in winter (Yuan et al., 2024). The timely bud dormancy and release are important for perennial plants to overwinter and grow normally in the next year. Therefore, a full understanding of the regulatory mechanisms of bud dormancy release is of great significance for plants. In this study, we found that a RING-H2 protein, PsATL33, played an important role in the regulation of tree peony bud dormancy. PsATL33 mainly modulated the accumulation of GAs, thereby functioning as a positive regulator of bud dormancy release.

Current studies have shown that the RING-H2 proteins are involved in a variety of physiological processes in plants. StATL2-like protein from *Solanum tuberosum* regulated plant growth and acted as a negative regulator of low-temperature tolerance (Song et al., 2022). PtXERICO from *Populus trichocarpa* improved plant drought tolerance by regulating the expression of ABA synthesis- and drought-related genes (Kim et al., 2020). IbATL38 reduced the accumulation of H_2_O_2_ to improve the salt tolerance of *Ipomoea batatas* plants (Du et al., 2021). The grape VpRH2 conferred enhanced resistance to powdery mildew (Wang et al., 2017a). MdCIP8 modulated anthocyanin accumulation to affect the apple plant response to light, thus contributing to hypocotyl elongation (Kang et al., 2020). In rice, OsMAL was involved in the accumulation of cytokinins and reactive oxygen species for promoting root development (Jiang et al., 2020). However, few studies have been reported on the regulation of RING-H2 proteins in bud dormancy. Here, we found that PsATL33, a member of the ATL family, participated in the modulation of bud dormancy. Heterologous expression of *PsATL33* in petunia promoted seed germination, plant growth, and dormancy release of axillary buds. Silencing of *PsATL33* in tree peony buds resulted in delayed bud germination, shown as a significant decrease in bud break rate and plant height. The results supported that PsATL33 serves as a positive regulator of bud dormancy release.

GAs and ABA are two important hormones during the bud dormancy transition. These two hormones have opposite effects on the dormancy process (Zhuang et al., 2013). Overproduction of GAs promotes the dormancy release of plants, and dormancy maintenance depends on high levels of ABA (Howe et al., 2015; Wen et al., 2016; Khalil-Ur-Rehman et al., 2019; Yang et al., 2020). Several studies have shown that GAs promoted bud germination and growth of tree peony buds, while ABA treatment inhibited germination (Zheng et al., 2009; Guan et al., 2019). In our studies, we found that both GA and ABA treatments caused a significant change in the expression of *PsATL33*, with the effect of GAs being more significant. However, in *PsATL33*-silenced buds, there was no significant change in the accumulation of ABA. The production of bioactive GAs, GA_1_ and GA_3_, decreased in the buds with *PsATL33* silencing, and notably, GA_3_ content decreased more significantly. Therefore, we speculate that PsATL33 probably regulated tree peony bud dormancy by mediating the GA_3_ biosynthesis. Through expression assessment in *PsATL33*-silenced buds, it was found that a few GA biosynthesis-related genes, *PsGA20ox1*, *PsGA3ox1*, and *PsGA3ox3*, were significantly downregulated, and transcript abundances of *PsGA2ox1* and *PsGAI1A* involved in GA catabolism and signal transduction were elevated. Based on these data, we conclude that PsATL33 may regulate GA-induced bud dormancy release by targeting both GA biosynthesis and signaling pathways.

Increasing evidence has demonstrated that ATL proteins may help plants adapt to environmental stresses through ubiquitin-mediated protein degradation (Ariani et al., 2016). *Arabidopsis* AtATL2, rice OsATL5, and potato StRFP1 were reported to be directly involved in the defense against external stresses (Salinas-Mondragón et al., 1999; Takai et al., 2002; Ni et al., 2010). LeATL6 from *Lycopersicon esculentum* played an important role in defense response by participating in the regulation of JA signaling (Hondo et al., 2007). ATL78 affected the sensitivity of plants to ABA and participated in the response of *Arabidopsis* plants to drought stress (Suh et al., 2016). GmRFP1 was also involved in stress responses via ABA signaling in soybean (Du et al., 2010). Moreover, *PsATL33* was found to be induced by ETH, MeJA, drought, salinity, and freezing treatments. We hypothesize that bud dormancy release may also be regulated by other plant hormones apart from GAs and ABA. It has been reported that a subset of ETH pathway-related genes were potentially implicated in the regulation of bud dormancy in grape (Shi et al., 2018). A recent report revealed that transcription factors BZR2/MYC2 regulated pear bud dormancy by modulating JA signaling (Wang et al., 2023).

In addition, PsATL33 may play a crucial role in plant tolerance to abiotic stresses, which was validated by our freezing assay (**Supplementary Figure S4**). The results showed that the buds inoculated with TRV-*PsATL33* exhibited a reduced tolerance to freezing stress (−4°C) compared with the control, suggesting that PsATL33 positively regulates the tolerance of tree peony buds to low temperatures. It has been demonstrated that some relationship between bud dormancy and cold acclimation exists in plants (Li et al., 2003b). However, bud dormancy and freezing tolerance can be regulated independently (Rinne et al., 1997), and their molecular mechanisms are different to a large extent. Our findings revealed an important role of PsATL33 in the regulation of both bud dormancy and cold acclimation. The specific mechanism for PsATL33’s role in cold acclimation remains still elusive. Although it is known that tree peony buds can endure extremely low temperature in winter, the tolerance can be largely decreased when the dormancy is fully released, and notably, bud break occurs (Vitra et al., 2017). The young germinating buds of tree peony are easily damaged by occasional freezing stress in early spring. Thus, the temperature (−4°C) we used in this assay can be considered serious stress at the stage of bud dormancy release and break in tree peony. Moreover, considering the upregulation of *PsATL33* by drought and salinity, a further investigation of the role of PsATL33 in the tolerance to these two stresses should be performed in future work.

## Data availability statement

The datasets presented in this study can be found in online repositories. The names of the repository/repositories and accession number(s) can be found in the article/**Supplementary material**.

## Author contributions

YM: Conceptualization, Data curation, Investigation, Methodology, Writing – original draft. YY: Data curation, Investigation, Methodology, Writing – original draft. YG: Data curation, Investigation, Writing – original draft. LZ: Data curation, Investigation, Writing – original draft. SF: Data curation, Investigation, Writing – original draft. JL: Writing – review & editing. DS: Conceptualization, Funding acquisition, Methodology, Supervision, Writing – original draft, Writing – review & editing.
