# Supplementary material for: A tree peony RING-H2 finger protein, PsATL33, plays an essential role in cold-induced bud dormancy release by regulating gibberellin content
Source: Front Plant Sci. 2024 Jun 3;15:1395530. doi: 10.3389/fpls.2024.1395530 (PMC11180761; doi:10.3389/fpls.2024.1395530)
Supplement: Supplementary file 1 [file DataSheet_1.docx]

**
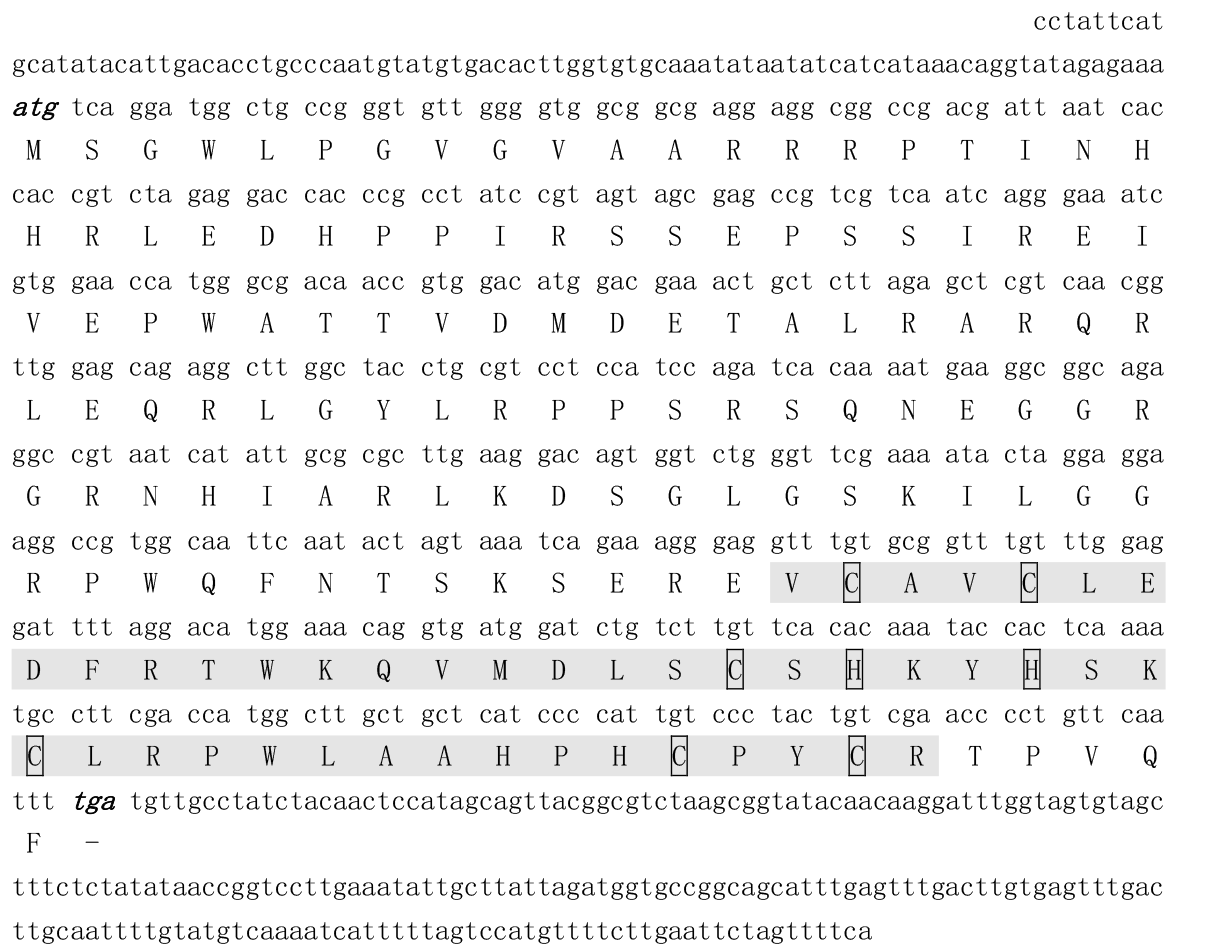
**

**Supplementary Figure S1.** **The nucleotide and deduced amino acid sequences of *PsATL33*’s cDNA.** The cDNA sequence of *PsATL33* contains a 483-bp coding region encoding a polypeptide of 161 amino acids. The italic bold fonts indicate the start and stop codons. The fonts shaded in grey indicate the RING-H2 conserved domain. The key cysteine (C) and histidine (H) residues are boxed.

**
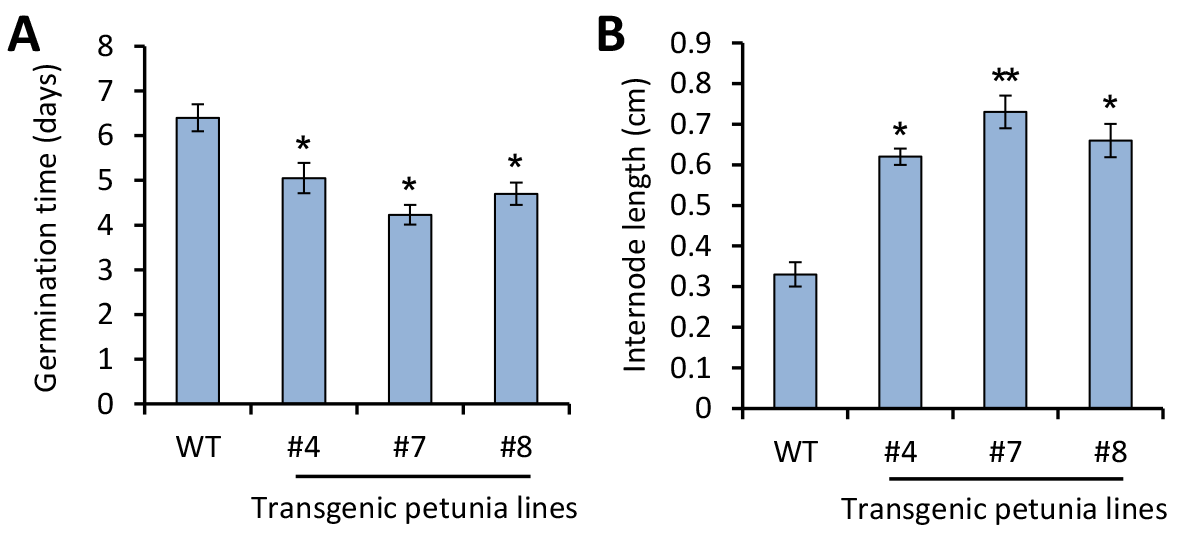
**

**Supplementary Figure S2. Overexpression of *PsATL33* accelerates seed germination and increases internode length in petunia.** (A) Germination time of the seeds from WT and transgenic petunia lines (#4, #7, and #8). Germination time was determined as the duration from seed sowing to seedling emerging from the soil. (B) Internode length of transgenic petunia lines in comparison to WT plants. The plants at 40 days after sowing were used for measurement of internode length. Error bars represent standard error of the mean from three biological replicates. Asterisks indicate statistical significance as determined by Student’s *t* test (^∗^*P* < 0.05, ^∗∗^*P* < 0.01).

**
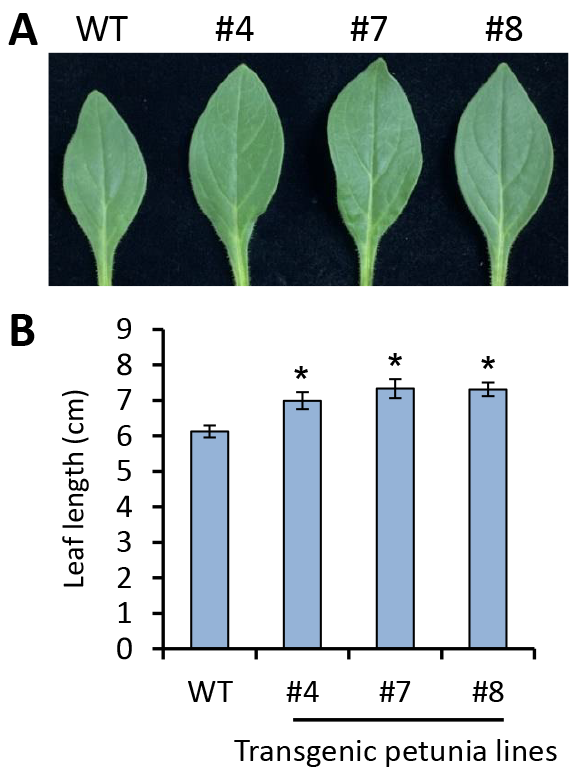
**

**Supplementary Figure S3. Overexpression of *PsATL33* increases leaf size in petunia.** (A) Representative phenotypes of leaves from wild-type (WT) and *PsATL33*-overexpressing transgenic petunia lines (#4, #7, and #8). The sixth leaves from the terminal of petunia plants at 40 days after sowing were photographed. (B) Leaf length of WT and transgenic petunia plants. Error bars represent standard error of the mean from three biological replicates. Asterisks indicate statistical significance as determined by Student’s *t* test (^∗^*P* < 0.05).


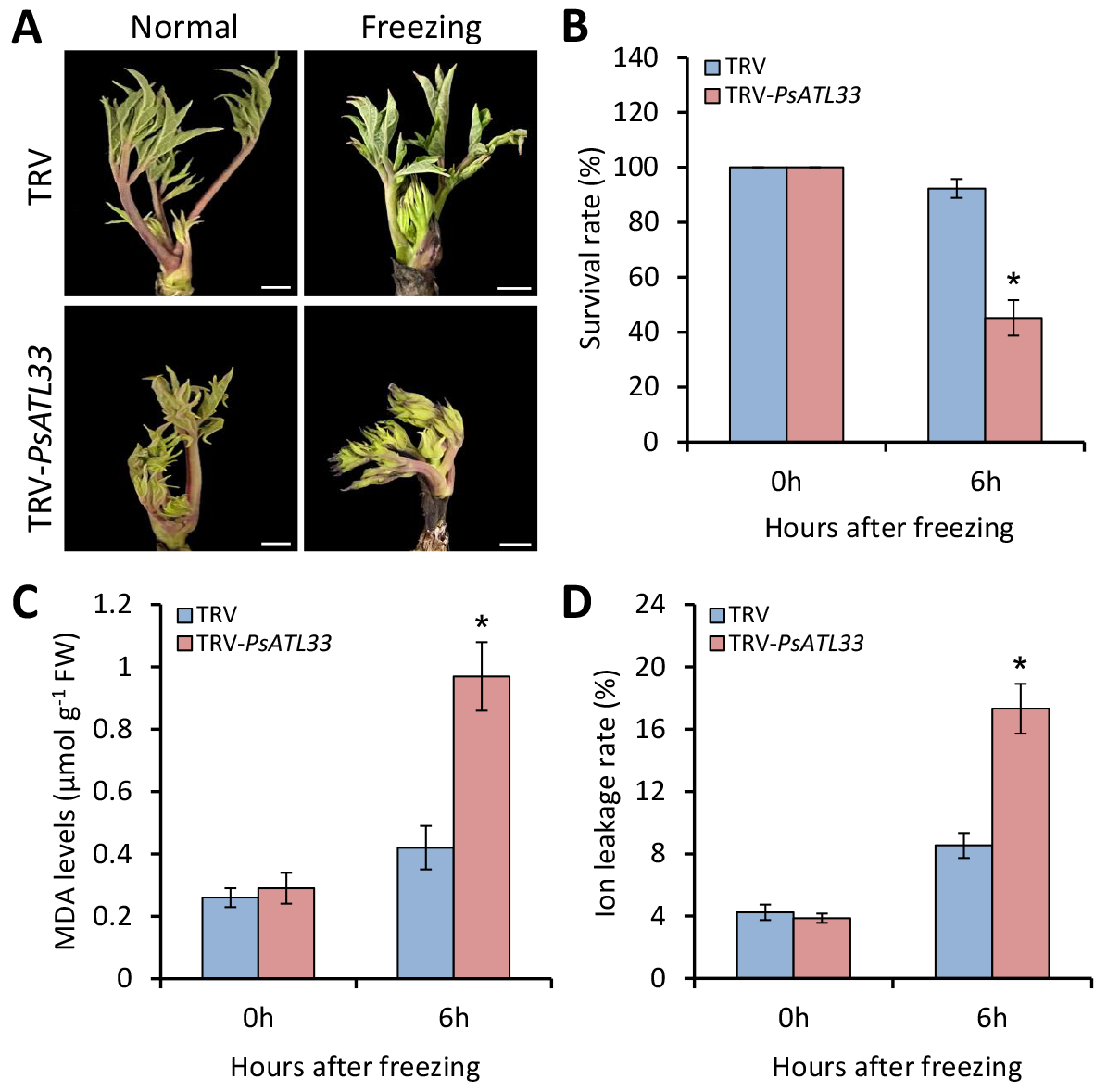


**Supplementary Figure S4.** **Silencing of *PsATL33* reduces bud tolerance to freezing stress.** (A) Representative symptoms of tree peony buds inoculated with TRV empty vector and TRV-*PsATL33* upon exposure to normal condition (22^o^C) and freezing stress (-4^o^C). Photographs were taken at 6 h after freezing treatment. (B) Survival rate of tree peony buds inoculated with different TRV constructs at 0 and 6 h after freezing treatment. Malondialdehyde (MDA) content (C) and ion leakage rate (D) in the buds after freezing treatment at intervals. Error bars represent standard error of the mean from three biological replicates. Asterisks indicate statistical significance as determined by Student’s *t* test (^∗^*P* < 0.05).

**Supplementary Table S1. Specific primers used for expression analysis and plasmid construct.**

| Gene name | Forward primer (5’-3’) | Reverse primer (5’-3’) | Purpose |
| --- | --- | --- | --- |
| *PsATL33* | TGCGGTTTGTTTGGAGGATTT | GTTGTATACCGCTTAGACGCC | RT-qPCR |
| *PsATL33* | GGTACCATGTCAGGATGGCTGCCGGGT | GGATCCAAATTGAACAGGGGTTCGA | Subcellular localization |
| *PsATL33* | GGTACCTTAATCACCACCGTCTAGAGG | CTCGAGACAAGACAGATCCATCACCTG | TRV-VIGS |
| *PsATL33* | GGATCCATGTCAGGATGGCTGCCGGGT | GTCGACTCAAAATTGAACAGGGGTTCG | Petunia transformation |
| *PsEBB1* | CGCCAAACACCATTTCCTCA | GCCTCATATCGTCCTGTCCA | RT-qPCR |
| *PsCXE15* | GCATTGGATCGAGGTTCACC | GAATTGGCTCCGGTTTGTGT | RT-qPCR |
| *PsKO* | TTCTCAAATGGGCAGTTTGTC | CTTTGACAACATCGACGGAAT | RT-qPCR |
| *PsKAO1* | AAAGGGTTGACGCTTAAGGAA | GTCATCCCATCTGGAAGGATT | RT-qPCR |
| *PsGA20ox1* | CGAGAACTAGCGGTTCCTCTT | ACTTGCATACCCACAATGCTC | RT-qPCR |
| *PsGA3ox1* | TGAAGAGCCTAGCAGAACGAG | GACGAACTGACACCCAACCTA | RT-qPCR |
| *PsGA3ox3* | TAATCCAATCCTCTTCATCTT | CCATAAGGTCCACAACAGGT | RT-qPCR |
| *PsGA2ox1* | TCAACCAAGAAATGGGTTCAG | CTGCAAGGAGTCACCAACATT | RT-qPCR |
| *PsGID1A* | CCAACTGCCAGTCTTGAACAA | GGACTGGTACTGGAGAGCTTT | RT-qPCR |
| *PsGAI1A* | CTCAACGCCAATTGTTTCAGC | GCCACCAGAGTTCATGTGATC | RT-qPCR |
| *PsRGL1* | TCAGAACTAGAACTCCGGAGC | GATAACTTGCCTTGACCCACC | RT-qPCR |
| *PsActin* | ATACTCATCCTCCACCACCAC | GGTATTGCCGATCGTATGAGC | RT-qPCR |
| *PhEF1α* | CCTGGTCAAATTGGAAACGG | CAGATCGCCTGTCAATCTTGG | RT-qPCR |
